# Glucocorticoid Receptor Binding Inhibits an Intronic *IL33* Enhancer and is Disrupted by rs4742170 (T) Allele Associated with Specific Wheezing Phenotype in Early Childhood

**DOI:** 10.3390/ijms19123956

**Published:** 2018-12-09

**Authors:** Alisa M. Gorbacheva, Dmitry V. Kuprash, Nikita A. Mitkin

**Affiliations:** 1 Laboratory of Intracellular Signaling in Health and Disease, Engelhardt Institute of Molecular Biology, Russian Academy of Sciences, 119991 Moscow, Russia; alisamur93@mail.ru (A.M.G.); kuprash@gmail.com (D.V.K.); 2 Biological Faculty, Lomonosov Moscow State University, 119234 Moscow, Russia

**Keywords:** asthma, allergy, wheezing, glucocorticoids, lung epithelium, inflammation

## Abstract

Interleukin 33 (IL-33) is a cytokine constitutively expressed by various cells of barrier tissues that contribute to the development of inflammatory immune responses. According to its function as an alarmin secreted by lung and airway epithelium, IL-33 plays a significant role in pathogenesis of allergic disorders. IL-33 is strongly involved in the pathogenesis of asthma, anaphylaxis, allergy and dermatitis, and genetic variations in *IL33* locus are associated with increased susceptibility to asthma. Genome-wide association studies have identified risk “T” allele of the single-nucleotide polymorphism rs4742170 located in putative *IL33* enhancer area as susceptible variant for development of specific wheezing phenotype in early childhood. Here, we demonstrate that risk “T” rs4742170 allele disrupts binding of glucocorticoid receptor (GR) transcription factor to *IL33* putative enhancer. The *IL33* promoter/enhancer constructs containing either 4742170 (T) allele or point mutations in the GR-binding site, were significantly more active and did not respond to cortisol in a pulmonary epithelial cell line. At the same time, the constructs containing rs4742170 (C) allele with a functional GR-binding site were less active and further inhibitable by cortisol. The latter effect was GR-dependent as it was completely abolished by GR-specific siRNA. This mechanism may explain the negative effect of the rs4742170 (T) risk allele on the development of wheezing phenotype that strongly correlates with allergic sensitization in childhood.

## 1. Introduction

IL-33 belongs to the family of IL-1 cytokines and is mainly expressed by a wide range of non-hematopoietic cells, such as epithelial cells, endothelial cells, myofibroblasts and fibroblast-like cells during homeostasis and in inflammatory conditions [1,2,3]. It also functions as a tissue-derived environmental alarmin that is released in response to infectious agents, allergens or injury [4]. Secreted IL-33 binds to heterodimer receptor complex consisting of interleukin 1 receptor accessory proteins (IL-1RAcP) and interleukin 1 receptor-like 1 (IL1RL1) protein expressed on the surface of various immune cells [1,5]. IL-33 targets many cell types that participate in allergic inflammation, including type 2 helper T cells, eosinophils, basophils, alternatively activated macrophages, dendritic cells, mast cells and type-2 innate lymphoid cells (ILC2) [4,6,7]. Via binding to receptor, IL-33 mediates cell activation, direct migration and increased production of classic Th2 cytokines, such as IL-4, IL-5 and IL-13 [8,9]. Recent data indicate that ILC2 cells producing large amounts of these cytokines in response to IL-33 play a prominent role in driving the systemic type-2 immune response and in the development of various allergic diseases [4,6,7,10].

Thus, IL-33 is involved in regulation of type 2 inflammation that is known to be the dominant mechanism in asthma [11]. Numerous studies have shown that local administration or lung specific transgenic expression of IL-33 is sufficient to provoke various asthma-associated symptoms, including inflammatory cellular infiltration, airway hyperresponsiveness and remodeling, collagen disposition, airway smooth muscle hypertrophy, goblet cell hyperplasia, increased pulmonary vascularity and tissue damping [12,13,14]. Elevated *IL33* mRNA expression has been detected in airway epithelium [15] and in cells derived from the sputum of asthmatic patients [16], whereas increased IL-33 protein was also observed in bronchoalveolar lavage fluid (BALF) [15,17], serum specimens [16], primary lung mast cells, airway smooth muscle and bronchial epithelium [18]. It is worth noting that in most cases IL-33 levels demonstrate a correlation with the exacerbation of asthma symptoms. 

Interestingly, multiple genetic studies demonstrated the association of numerous single nucleotide polymorphisms (SNPs) in the *IL33* locus with asthma susceptibility [19,20,21,22] and even with formation of certain phenotypes of respiratory diseases [20]. In particular, “T” allele of the polymorphism rs4742170 located in the second intron of *IL33* gene was linked to specific wheezing phenotype (intermediate-onset wheeze) [23]. According to results by Savenije et al., this wheezing phenotype is closely associated with allergic sensitization in childhood and presumably could affect subsequent asthma development [23].

Based on the above data, we hypothesized that rs4742170 “T” allele could affect *IL33* transcription leading to elevated expression of *IL33* gene. Experimental confirmation of rs4742170 functional significance for the *IL33* transcriptional regulation would improve the understanding of IL-33 biology in the context of allergy. In this paper, we show that the presence of rs4742170 “T” allele in a putative *IL33* enhancer correlates with increased activity of the *IL33* promoter due to destruction of the overlapping GR-binding site. Our results suggest an explanation for association of rs4742170 with the development of specific wheezing phenotype in children.

## 2. Results

### 2.1. A Putative Enhancer Including rs4742170 Stimulates IL33 Promoter Activity

**Figure 1:**
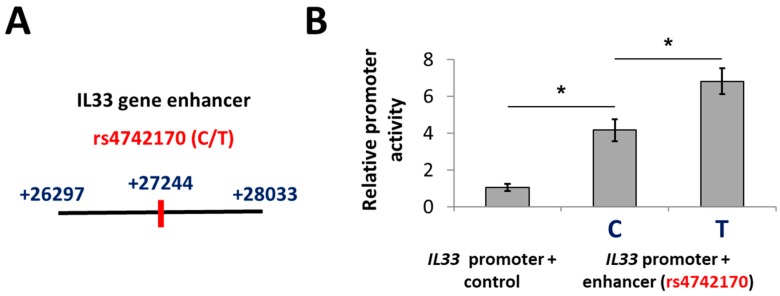
Predicted enhancer including rs4742170 induces *IL33* promoter activity in a luciferase reporter system. (**A**) Schematic illustration of rs4742170 location in *IL33* enhancer. (**B**) *IL33* enhancer containing rs4742170 (T) allele stimulated *IL33* promoter activity in lung cancer cells stronger than the rs4742170 (C) variant. The experiment was performed for five times. Relative promoter activities were calculated by normalization of Firefly luciferase signals to *Renilla* luciferase activities with further normalization to “*IL33* promoter + control” signal. * *p* < 0.01.

Single-nucleotide polymorphism rs4742170 is located in the second intron of the human *IL33* gene, more than 27 kb from the distal *IL33* promoter characterized in our previous work [24] and +27244 bp from the TSS. Based on the available epigenetic data [25], the 5′ part of intron 2 of the *IL33* gene containing rs4742170 has a number of features typical for regulatory elements (Supplementary Figure S1). For functional evaluation, we designated the region from +26297 to +28033 bp from TSS a putative enhancer and cloned it into a luciferase reporter vector (Figure 1A). We supplemented the luciferase reporter vector already containing the *IL33* promoter [24] with different variants of a downstream enhancer: an irrelevant control fragment without enhancer properties; enhancer with protective (C) allele of rs4742170; and enhancer with risk (T) allele of rs4742170. The activities of these constructs were compared in NCIH-196 human lung carcinoma cell line [24]. Both versions of the putative enhancer strongly increased the *IL33* promoter activity in this reporter system, with an additional significant effect of the risk rs4742170 (T) allele (Figure 1B).

### 2.2. The rs4742170 (T) Allele in IL33 Enhancer Disrupts GR-Binding Site

In order to see what specific transcription factor binding sites (TFBS) in the *IL33* enhancer may be affected by the rs4742170 polymorphism, we applied PERFECTOS-APE software [26] with TFBS models from the HOCOMOCO v11 collection. The resulting list of candidate transcription factors with the best scores included basic helix-loop-helix proteins OLIG2 and LYL1, nuclear receptors for androgen (AR) and glucocorticoids (GR) and an HMG-box protein SOX10. Interestingly, binding of all these factors was predicted to be strongly reduced in the presence of the rs4742170 (T) allele. To the best of our knowledge, none of these factors except GR has been reported to be involved in asthma pathogenesis. In particular, OLIG2 has been shown to play a key role in oligodendrocyte differentiation [27,28]; LYL1 is known in relation to T cell leukaemogenesis [29] and hematopoiesis [30]; AR participates in sexual differentiation [31] and is central to the development and treatment of prostate cancer [32]; SOX10 has been implicated in the regulation of embryonic development [33] and in proliferation and survival of hematopoietic stem cells and hematopoietic tumors [34,35]. As for the GR, it is well known to regulate many steroid-responsive genes participating in stress response and inflammation [36] via its association with glucocorticoid response elements [37]. High levels of cytosolic GR are constitutively expressed by lung epithelium [38], GR activity, and nuclear translocation is triggered by binding to cortisol [39]. Glucocorticoids are widely used for the treatment of asthma and allergic disorders [40], contributing to suppression of airway hyper-responsiveness, reduction of airway edema and infiltration of inflammatory cells from the blood to the airways [41]. Apparently, their therapeutic effects are mostly mediated by the ability to inhibit the expression of inflammatory genes through various DNA-binding mechanisms [42,43,44,45]. However, available data on the specific GR-DNA interactions in the GR target genes are rather limited and poorly defined [44,46,47]. We hypothesized that risk rs4742170 (T) allele could interfere with such an interaction in human *IL33* locus.

**Figure 2:**
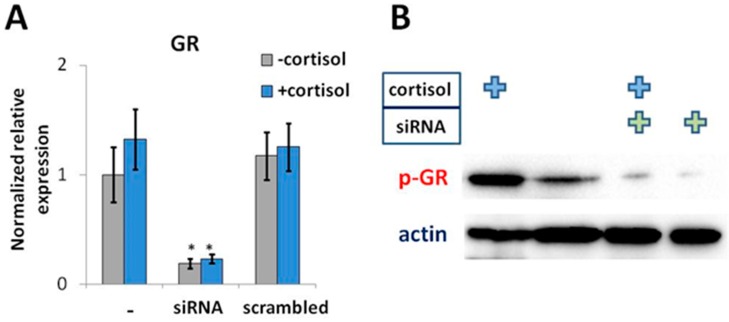
Cortisol-mediated activation promotes hyperphosphorylation of GR in NCIH-196 lung cancer cells. Stimulation by cortisol (50 ng/mL, 24 h) did not influence the level of *GR* expression (**A**) but resulted in elevated levels of phosphorylated GR protein (**B**). “siRNA” and “scrambled” indicate transfection with specific siRNA or control scrambled siRNA, respectively. Real-time PCR data was obtained using the ΔΔCt approach, normalized to β-actin and represented as Mean ± SEM (five independent experiments). * *p* < 0.01. Western blot data is a representative image of three replicate experiments.

Treatment with cortisol for 24 h did not affect the level of *GR* mRNA in NCIH-196 cells (Figure 2A) while GR phosphorylation that is required for the translocation of activated GR–steroid complex into the nucleus [48] was significantly induced (Figure 2B). Administration of specific siRNA led to a significant decrease in both *GR* mRNA and phosphorylated GR (p-GR), regardless of experimental conditions (Figure 2A,B).

**Figure 3:**
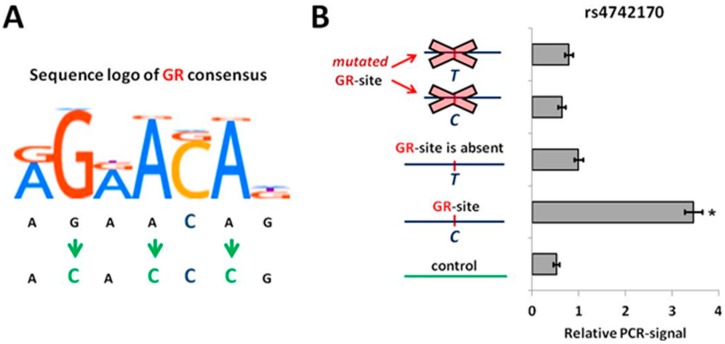
Risk “T” allele of rs4742170 disrupts GR-binding site in *IL33* enhancer. (**A**) Position weight matrix of GR-binding site and the scheme of its point mutagenesis. (**B**) The level of GR binding to rs4742170 alleles was estimated by pull-down assay with nuclear extracts from cortisol-stimulated NCIH-196 lung cancer cells. The results of three independent experiments are represented. * *p* < 0.01.

In order to evaluate the influence of rs4742170 alleles on GR binding to *IL33* enhancer, we performed a pull-down assay using nuclear extracts from NCIH-196 cells pre-treated with cortisol to stimulate GR nuclear translocation. We amplified four variants of a 200 bp *IL33* enhancer fragment flanking the rs4742170 (+27776 to +27976 from TSS) containing combinations of the rs4742170 alleles with either intact or GR TFBS (Figure 3A). The probe containing the rs4742170 (C) allele demonstrated high levels of precipitation with anti-GR antibodies, whereas the presence of either the risk “T” allele or the GR-site point mutations was associated with much lower levels of precipitation comparable to background signal observed with control probe (Figure 3B). Thus, the rs4742170 (T) allele actually creates a critical mutation of the functional GR TFBS in the *IL33* enhancer.

### 2.3. GR Activation is Associated with a Decrease in IL33 Promoter Activity

**Figure 4:**
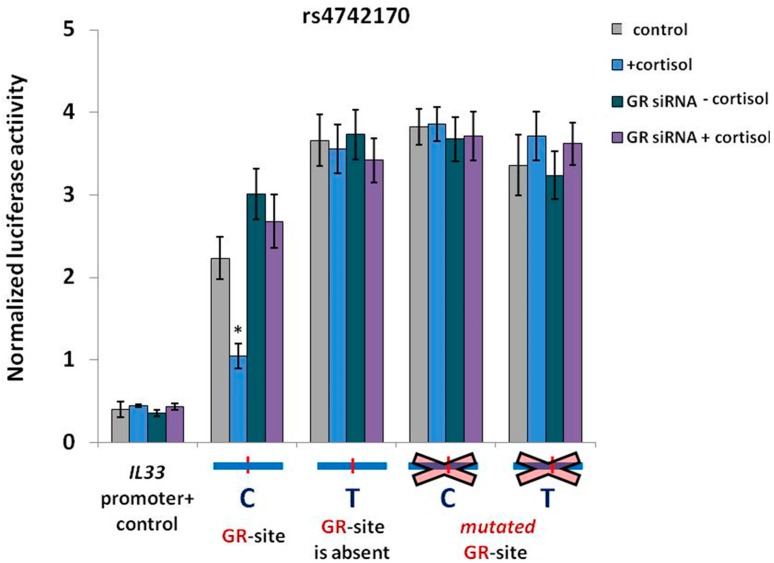
Reduced binding of GR to *IL33* enhancer correlated with elevated *IL33* promoter activity in NCIH-196 lung cancer cells. Reporter vectors included *Firefly* luciferase gene under *IL33* promoter containing one of the SNP rs4742170 variants. Cortisol (50 ng/mL) treatment was performed 24 h before and during electroporation to stimulate GR activity. GR-specific siRNA was added 24 h prior to electroporation for *GR* knockdown. The data shown (mean ± SEM) was obtained in five independent experiments and normalized to *Renilla* luciferase activity. * *p* < 0.01.

In order to identify the role of GR in regulation of the *IL33* promoter activity, we used reporter vectors containing the *IL33* promoter and an enhancer bearing one of the rs4742170 versions tested in the DNA binding experiments. We performed luciferase reporter assays under normal conditions, in the context of cortisol-induced stimulation or upon siRNA-mediated GR suppression. Cortisol-mediated GR induction affected the *IL33* promoter activity only in the presence of the enhancer element carrying the functional GR-binding site (Figure 4). The risk 4742170 (T) allele in *IL33* enhancer, as well as point mutations of the GR-binding site, increased the basal activity of the reporter constructs and made them completely unresponsive to cortisol. At the same time, the activity of the *IL33* promoter construct containing the common rs4742170 (C) allele with functional GR-binding site was lower and went further down upon cortisol treatment. The effect of the cortisol-induced GR stimulation with subsequent suppression of *IL33* promoter activity was abolished by the addition of GR-specific siRNA. These results suggest the negative role of the risk rs4742170 (T) allele in asthma may be mechanistically related to the increased *IL33* promoter activity, due to reduced binding of the GR transcription factor.

## 3. Discussion

As already noted above, effective application of glucocorticoid hormones to the treatment of chronic respiratory diseases is mainly attributed to the ability of the GR transcription factor to reduce expression of proinflammatory genes [40]. The list of inflammatory mediators that can be suppressed by glucocorticoids includes IL-5, IL-13, TNF, IL-6, TSLP, and others [49]. IL-33 is an attractive candidate for this list since its elevated expression in lung bronchial epithelium is thought to be crucial for the stabilization of inflammatory conditions and exacerbation of airway hyper-responsiveness and airway remodeling [50,51,52]. The latter involves pathological structural changes such as neoangiogenesis and increased reticular basement membrane thickness that may provoke an irreversible decrease in the airway lumen (and, consequently, in airflow) that often accompanies respiratory disease [51,52]. Nevertheless, the data on the influence of glucocorticoid hormones on IL-33 expression have been controversial, as in some cases IL-33 production was decreased by glucocorticoids [53] while other studies indicated its insensitivity to these hormones [50,51].

In our previous study [24] we revealed a possible molecular mechanism underlying the association between SNP rs928413 in the *IL33* promoter and elevated risk of asthma progression. We demonstrated that risk “G” rs928413 allele creates a CREB1 binding site that is sufficiently strong to provide a significant boost in *IL33* transcriptional activation in lung carcinoma cells. In the present study, we evaluated the functional significance of another SNP, rs4742170, associated with the development of another airway pathology, a specific wheezing phenotype. Of all known SNPs associated with asthma and allergic disease, rs4742170 is the only one to be located in a putative regulatory area of the *IL33* gene that is associated with binding of transcription factors relevant to the progression of airway pathologies. In particular, our analysis demonstrated that rs4742170 variation interferes with the binding of GR. Since the genomic region spanning the rs4742170 demonstrates a significant enhancer effect on the *IL33* promoter in a reporter gene assay, it may represent a mechanistic link of the rs4742170 polymorphism to intermediate-onset wheezing phenotype in early childhood. Enhanced secretion of IL-33 by respiratory epithelium could promote the smooth muscle contraction of the airway, airway hyper-responsiveness, and the aggravation of inflammatory response during allergic sensitization in childhood and presumably might affect subsequent asthma development. Such a mechanism would corroborate the established critical role of epithelium in the pathogenesis of allergic disorders [54,55]. Obviously, our experimental strategy has a number of methodological limitations that could influence the clinical relevance of the conclusions: the model system is a cultured cell line rather than live epithelium, regulatory elements of the *IL33* gene in the luciferase reporter vector are isolated from the genomic context and the TF binding is assessed by an in vitro test. On the other hand, these approaches make the model system more defined, simplify the interpretation of the results, and are widely used to study the regulation of human genes [56,57,58].

Interestingly, the rs4742170 “T” allele is abundant in various human populations, with an average frequency of 53.7% [59]. Thus, the molecular mechanism described in this study may contribute to excessive pathology in a variety of conditions amendable by glucocorticoid therapy. Therefore, correlations between *IL33* expression, rs4742170 allele variants, GR activity and clinical outcome would be an interesting parameter to monitor in clinical studies involving cortisol treatment.

## 4. Materials and Methods

### 4.1. Cell Lines

NCIH-196 human lung carcinoma cell line was received from American Type Culture Collection (ATCC, Manassas, VA, USA). Cells were cultured in RPMI-1640 medium (Life Technologies, Carlsbad, CA, USA) containing 10% fetal bovine serum. For activation, cortisol (50 ng/mL) was added to cell culture for 24h.

### 4.2. Ethical Approval

Scientific Council of Engelhardt Institute of Molecular Biology reported that no ethical approval is required for experiments performed in this study, because only commercially available cell lines were used.

### 4.3. Luciferase Reporter Constructs

Human *IL33* promoter reporter construct has been described [24]. *IL33* enhancer area, +26297 to +28033 from transcription start site (TSS), was amplified by PCR using genomic DNA of NCIH-196 cells and specific primers with BamHI/SalI restriction sites. *IL33* enhancer variants containing the rs4742170 (T) allele and mutations of the GR-binding site were amplified by overlapping PCR (see Supplementary Table S1 for sequences of all oligonucleotide primers). All *IL33* enhancer variants were cloned into BamHI/SalI sites of the *IL33* promoter reporter construct [24] based on pGL3-basic vector (Promega, Madison, WI, USA) and verified by Sanger sequencing. In order to equalize the overall plasmid size, the control *IL33* promoter reporter construct contained an irrelevant fragment of similar length from human *CXCR5* locus [60]. NCIH-196 cells transfection and measurement of the luciferase activity was performed as described [24].

### 4.4. Pull-Down Assay

We amplified 200-bp fragments of *IL33* enhancer region (+27776/+27976 bp from TSS) containing “C” or “T” rs4742170 allele with or without mutations in the GR-binding site using luciferase reporter constructs as templates. Our previously described control DNA fragment [24] did not contain any predictable GR-binding sites and was therefore used as a negative control here as well (see Supplementary Table S1 for PCR primers). NCIH-196 cells were pre-treated with cortisol prior to nuclear extraction to stimulate the translocation of the hormone-GR complex. Preparation of nuclear extracts, immunoprecipitation of DNA-protein complexes and quantification of bound DNA by real time PCR were described earlier [61]. We used rabbit polyclonal anti-GR antibodies (ab55189, Abcam, Cambridge, UK) to precipitate GR-DNA complexes. The background subtraction and data normalization was carried out using control binding reactions including rabbit IgG isotype control antiserum as described [24].

### 4.5. GR Knockdown Using siRNA

Transfection of NCIH-196 cells with GR-specific siRNA, RNA isolation, reverse transcription and measurement of *GR* mRNA level by RT-PCR were performed as described [62]. We used two published pairs of siRNAs targeting GR [63], one of which mediated a significant decrease in *GR* mRNA expression level in NCIH-196 cells and was chosen for the experiments. The sequences of siRNAs and GR-specific primers are indicated in Supplementary Table S1.

### 4.6. Western Blot Analysis

Standard protocols of protein sample collection, electrophoresis and transfer to nitrocellulose membrane were followed [24] using membrane pre-blocking with 5% non-fat dry milk, anti-GR antibodies at 1:2000 dilution, HRP-conjugated secondary anti-rabbit antibodies at 1:30,000 dilution and anti-β-actin antibodies (ab8229, Abcam, Cambridge, UK) as a loading control at 1:3000 dilution.

### 4.7. Statistical Analysis

We used Microsoft Excel for statistical analyses. Statistical significance was determined using two-tailed unpaired Student’s *t*-test. Data were represented as mean ± SEM.

## Supplementary Materials

Supplementary materials can be found at http://www.mdpi.com/1422-0067/19/12/3956/s1.

## References

[1] Moussion C., Ortega N., Girard J.-P. (2008). The IL-1-Like Cytokine IL-33 Is Constitutively Expressed in the Nucleus of Endothelial Cells and Epithelial Cells In Vivo: A Novel ‘Alarmin’?. PLoS ONE.

[2] Pichery M., Mirey E., Mercier P., Lefrancais E., Dujardin A., Ortega N., Girard J.-P. (2012). Endogenous IL-33 is highly expressed in mouse epithelial barrier tissues, lymphoid organs, brain, embryos, and inflamed tissues: In situ analysis using a novel Il-33-LacZ gene trap reporter strain. J. Immunol..

[3] Cayrol C., Girard J.-P. (2018). Interleukin-33 (IL-33): A nuclear cytokine from the IL-1 family. Immunol. Rev..

[4] Cayrol C., Girard J. (2014). IL-33: An alarmin cytokine with crucial roles in innate immunity, inflammation and allergy. Curr. Opin. Immunol..

[5] Lingel A., Weiss T.M., Niebuhr M., Pan B., Appleton B.A., Wiesmann C., Bazan J.F., Fairbrother W.J. (2009). Structure of IL-33 and Its Interaction with the ST2 and IL-1RAcP Receptors-Insight into Heterotrimeric IL-1 Signaling Complexes. Structure.

[6] Molofsky A.B., Savage A.K., Locksley R.M. (2015). Interleukin-33 in Tissue Homeostasis, Injury, and Inflammation. Immunity.

[7] Liew F.Y., Girard J.P., Turnquist H.R. (2016). Interleukin-33 in health and disease. Nat. Rev. Immunol..

[8] Smith D.E. (2009). IL-33: A tissue derived cytokine pathway involved in allergic inflammation and asthma. Clin. Exp. Allergy.

[9] Liew F.Y., Pitman N.I., McInnes I.B. (2010). Disease-associated functions of IL-33: The new kid in the IL-1 family. Nat. Rev. Immunol..

[10] Matsuki A., Takatori H., Makita S., Yokota M., Tamachi T., Suto A., Suzuki K., Hirose K., Nakajima H. (2016). T-bet inhibits innate lymphoid cell–mediated eosinophilic airway inflammation by suppressing IL-9 production. J. Allergy Clin. Immunol..

[11] Woodruff P.G., Modrek B., Choy D.F., Jia G., Abbas A.R., Ellwanger A., Arron J.R., Koth L.L., Fahy J.V. (2009). T-helper type 2-driven inflammation defines major subphenotypes of asthma. Am. J. Respir. Crit. Care Med..

[12] Kurowska-Stolarska M., Kewin P., Murphy G., Russo R.C., Stolarski B., Garcia C.C., Komai-Koma M., Pitman N., Li Y., McKenzie A.N.J. (2008). IL-33 Induces Antigen-Specific IL-5+ T Cells and Promotes Allergic-Induced Airway Inflammation Independent of IL-4. J. Immunol..

[13] Li Y., Wang W., Huang P., Zhang Q., Yao X., Wang J., Lv Z., An Y., Corrigan C.J., Huang K. (2015). Distinct sustained structural and functional effects of interleukin-33 and interleukin-25 on the airways in a murine asthma surrogate. Immunology.

[14] Kondo Y., Yoshimoto T., Yasuda K., Futatsugi-yumikura S., Morimoto M., Hayashi N., Hoshino T., Fujimoto J., Nakanishi K. (2008). Administration of IL-33 induces airway hyperresponsiveness and goblet cell hyperplasia in the lungs in the absence of adaptive immune system. Int. Immunol..

[15] Préfontaine D., Nadigel J., Chouiali F., Audusseau S., Semlali A., Chakir J., Martin J.G., Hamid Q. (2010). Increased IL-33 expression by epithelial cells in bronchial asthma. J. Allergy Clin. Immunol..

[16] Hamzaoui A., Berraies A., Kaabachi W., Haifa M., Ammar J., Kamel H. (2013). Induced sputum levels of IL-33 and soluble ST2 in young asthmatic children. J. Asthma.

[17] Liu S., Verma M., Michalec L., Liu W., Sripada A., Rollins D., Good J., Ito Y., Chu H.W., Gorska M.M. (2018). Steroid resistance of airway type 2 innate lymphoid cells from patients with severe asthma: The role of thymic stromal lymphopoietin. J. Allergy Clin. Immunol..

[18] Kaur D., Gomez E., Doe C., Berair R., Woodman L., Saunders R., Hollins F., Rose F.R., Amrani Y., May R. (2015). IL-33 drives airway hyper-responsiveness through IL-13-mediated mast cell: Airway smooth muscle crosstalk. Allergy Eur. J. Allergy Clin. Immunol..

[19] Gudbjartsson D.F., Bjornsdottir U.S., Halapi E., Helgadottir A., Sulem P., Jonsdottir G.M., Thorleifsson G., Helgadottir H., Steinthorsdottir V., Stefansson H. (2009). Sequence variants affecting eosinophil numbers associate with asthma and myocardial infarction. Nat. Genet..

[20] Moffatt M., Gut I., Demenais F., Strachan D., Bouzigon E., Heath S. (2010). A Large-Scale, Consortium-Based Genomewide Association Study of Asthma. N. Engl. J. Med..

[21] Torgerson D.G., Ampleford E.J., Chiu G.Y., Gauderman W.J., Gignoux C.R., Graves P.E., Himes B.E., Levin A.M., Mathias R.A., Hancock D.B. (2011). Meta-analysis of genome-wide association studies of asthma in ethnically diverse North American populations. Nat. Genet..

[22] Bonnelykke K., Sleiman P., Nielsen K., Kreiner-Moller E., Mercader J.M., Belgrave D., Den Dekker H.T., Husby A., Sevelsted A., Faura-Tellez G. (2014). A genome-wide association study identifies CDHR3 as a susceptibility locus for early childhood asthma with severe exacerbations. Nat. Genet..

[23] Savenije O.E., Mahachie John J.M., Granell R., Kerkhof M., Dijk F.N., De Jongste J.C., Smit H.A., Brunekreef B., Postma D.S., Van Steen K. (2014). Association of IL33-IL-1 receptor-like 1 (IL1RL1) pathway polymorphisms with wheezing phenotypes and asthma in childhood. J. Allergy Clin. Immunol..

[24] Gorbacheva A., Korneev K., Kuprash D., Mitkin N. (2018). The Risk G Allele of the Single-Nucleotide Polymorphism rs928413 Creates a CREB1-Binding Site That Activates IL33 Promoter in Lung Epithelial Cells. Int. J. Mol. Sci..

[25] Rosenbloom K.R., Sloan C.A., Malladi V.S., Dreszer T.R., Learned K., Kirkup V.M., Wong M.C., Maddren M., Fang R., Heitner S.G. (2013). ENCODE Data in the UCSC Genome Browser: Year 5 update. Nucleic Acids Res..

[26] Vorontsov I.E., Kulakovskiy I.V., Khimulya G., Nikolaeva D.D., Makeev V.J. PERFECTOS-APE: Predicting regulatory functional effect of SNPs by approximate P-value estimation. Proceedings of the International Conference on Bioinformatics Models, Methods and Algorithms.

[27] Zhou Q., Wang S., Anderson D.J. (2000). Identification of a novel family of oligodendrocyte lineage-specific basic helix-loop-helix transcription factors. Neuron.

[28] Takebayashi H., Yoshida S., Sugimori M., Kosako H., Kominami R., Nakafuku M., Nabeshima Y.I. (2000). Dynamic expression of basic helix-loop-helix Olig family members: Implication of Olig2 in neuron and oligodendrocyte differentiation and identification of a new member, Olig3. Mech. Dev..

[29] Mellentin J.D., Smith S.D., Cleary M.L. (1989). Lyl-1, a novel gene altered by chromosomal translocation in T cell leukemia, codes for a protein with a helix-loop-helix DNA binding motif. Cell.

[30] Souroullas G.P., Salmon J.M., Sablitzky F., Curtis D.J., Goodell M.A. (2009). Adult Hematopoietic Stem and Progenitor Cells Require Either Lyl1 or Scl for Survival. Cell Stem Cell.

[31] Brinkmann A.O., Trapman J. (2000). Genetic analysis of androgen receptors in development and disease. Adv. Pharmacol..

[32] Heinlein C.A., Chang C. (2004). Androgen receptor in prostate cancer. Endocr. Rev..

[33] Le Cam L., Lacroix M., Ciemerych M.A., Sardet C., Sicinski P. (2004). The E4F Protein Is Required for Mitotic Progression during Embryonic Cell Cycles. Mol. Cell. Biol..

[34] Chagraoui J., Niessen S., Lessard J., Girard S., Coulombe P., Meloche S., Sauvageau G. (2004). p120E4F-1: A novel candidate factor for mediating Bmi-1 function in hematopoietic stem cells. Blood.

[35] Hatchi E., Rodier G., Lacroix M., Caramel J., Kirsh O., Jacquet C., Schrepfer E., Lagarrigue S., Linares L.K., Lledo G. (2011). E4F1 deficiency results in oxidative stress-mediated cell death of leukemic cells. J. Exp. Med..

[36] Weikum E.R., Knuesel M.T., Ortlund E.A., Yamamoto K.R. (2017). Glucocorticoid receptor control of transcription: Precision and plasticity via allostery. Nat. Rev. Mol. Cell Biol..

[37] Chandler V.L., Maler B.A., Yamamoto K.R. (1983). DNA sequences bound specifically by glucocorticoid receptor in vitro render a heterologous promoter hormone responsive in vivo. Cell.

[38] Adcock I.M., Gilbey T., Gelder C.M., Chung K.F., Barnes P.J. (1996). Glucocorticoid receptor localization in normal and asthmatic lung. Am. J. Respir. Crit. Care Med..

[39] Funder J.W. (1997). Glucocorticoid and Mineralocorticoid Receptors: Biology and Clinical Relevance. Annu. Rev. Med..

[40] Barnes P.J. (2012). New drugs for asthma. Semin. Respir. Crit. Care Med..

[41] Caramori G., Adcock I. (2003). Pharmacology of airway inflammation in asthma and COPD. Pulm. Pharmacol. Ther..

[42] Rhen T., Cidlowski J.A. (2005). Antiinflammatory Action of Glucocorticoids—New Mechanisms for Old Drugs. N. Engl. J. Med..

[43] Newton R., Leigh R., Giembycz M.A. (2010). Pharmacological strategies for improving the efficacy and therapeutic ratio of glucocorticoids in inflammatory lung diseases. Pharmacol. Ther..

[44] Surjit M., Ganti K.P., Mukherji A., Ye T., Hua G., Metzger D., Li M. (2011). Widespread Negative Response Elements Mediate Direct Repression by Agonist- Liganded Glucocorticoid Receptor. Cell.

[45] Uhlenhaut N.H., Barish G.D., Yu R.T., Downes M., Karunasiri M., Liddle C., Schwalie P., Hübner N., Evans R.M. (2013). Insights into Negative Regulation by the Glucocorticoid Receptor from Genome-wide Profiling of Inflammatory Cistromes. Mol. Cell.

[46] De Bosscher K., Haegeman G. (2009). Minireview: Latest Perspectives on Antiinflammatory Actions of Glucocorticoids. Mol. Endocrinol..

[47] Reddy T.E., Pauli F., Sprouse R.O., Neff N.F., Newberry K.M., Garabedian M.J., Myers R.M. (2009). Genomic determination of the glucocorticoid response reveals unexpected mechanisms of gene regulation. Genome Res..

[48] Beato M., Herrlich P., Schütz G. (1995). Steroid hormone receptors: Many Actors in search of a plot. Cell.

[49] Barnes P.J. (2016). Glucocorticosteroids. Handbook of Experimental Pharmacology.

[50] Saglani S., Lui S., Ullmann N., Campbell G.A., Sherburn R.T., Mathie S.A., Denney L., Bossley C.J., Oates T., Walker S.A. (2013). IL-33 promotes airway remodeling in pediatric patients with severe steroid-resistant asthma. J. Allergy Clin. Immunol..

[51] Li Y., Wang W., Lv Z., Li Y., Chen Y., Huang K., Corrigan C.J., Ying S. (2018). Elevated Expression of IL-33 and TSLP in the Airways of Human Asthmatics In Vivo: A Potential Biomarker of Severe Refractory Disease. J. Immunol..

[52] Ding W., Zou G., Zhang W., Lai X., Chen H., Xiong L. (2018). Interleukin-33: Its Emerging Role in Allergic Diseases. Molecules.

[53] Nabe T., Wakamori H., Yano C., Nishiguchi A., Yuasa R., Kido H., Tomiyama Y., Tomoda A., Kida H., Takiguchi A. (2015). Production of interleukin (IL)-33 in the lungs during multiple antigen challenge-induced airway inflammation in mice, and its modulation by a glucocorticoid. Eur. J. Pharmacol..

[54] Lambrecht B.N., Hammad H. (2012). The airway epithelium in asthma. Nat. Med..

[55] Mitchell P.D., O’Byrne P.M. (2016). Biologics and the lung: TSLP and other epithelial cell-derived cytokines in asthma. Pharmacol. Ther..

[56] Niu N., Wang L. (2015). In vitro human cell line models to predict clinical response to anticancer drugs. Pharmacogenomics.

[57] He H., Li W., Liyanarachchi S., Srinivas M., Wang Y., Akagi K., Wang Y., Wu D., Wang Q., Jin V. (2015). Multiple functional variants in long-range enhancer elements contribute to the risk of SNP rs965513 in thyroid cancer. Proc. Natl. Acad. Sci. USA.

[58] Carey M.F., Peterson C.L., Smale S.T. (2012). Confirming the functional importance of a protein-DNA interaction. Cold Spring Harb. Protoc..

[59] The 1000 Genomes Project Consortium (2015). A global reference for human genetic variation. Nature.

[60] Mitkin N.A., Hook C.D., Schwartz A.M., Biswas S., Kochetkov D.V., Muratova A.M., Afanasyeva M.A., Kravchenko J.E., Bhattacharyya A., Kuprash D.V. (2015). p53-dependent expression of CXCR5 chemokine receptor in MCF-7 breast cancer cells. Sci. Rep..

[61] Mitkin N.A., Muratova A.M., Schwartz A.M., Kuprash D.V. (2016). The A allele of the Single-Nucleotide Polymorphism rs630923 creates a Binding site for MEF2C resulting in reduced CXCR5 Promoter activity in B-cell lymphoblastic cell lines. Front. Immunol..

[62] Mitkin N.A., Muratova A.M., Korneev K.V., Pavshintsev V.V., Rumyantsev K.A., Vagida M.S., Uvarova A.N., Afanasyeva M.A., Schwartz A.M., Kuprash D.V. (2018). Protective C allele of the single-nucleotide polymorphism rs1335532 is associated with strong binding of Ascl2 transcription factor and elevated CD58 expression in B-cells. Biochim. Biophys. Acta Mol. Basis Dis..

[63] Hasson S.A., Kane L.A., Yamano K., Huang C.H., Sliter D.A., Buehler E., Wang C., Heman-Ackah S.M., Hessa T., Guha R. (2013). High-content genome-wide RNAi screens identify regulators of parkin upstream of mitophagy. Nature.

