# Supplementary material for: Glucocorticoid Receptor Binding Inhibits an Intronic IL33 Enhancer and is Disrupted by rs4742170 (T) Allele Associated with Specific Wheezing Phenotype in Early Childhood
Source: Int J Mol Sci. 2018 Dec 9;19(12):3956. doi: 10.3390/ijms19123956 (PMC6321062; doi:10.3390/ijms19123956)
Supplement: Supplementary file 1 [file ijms-19-03956-s001.pdf]

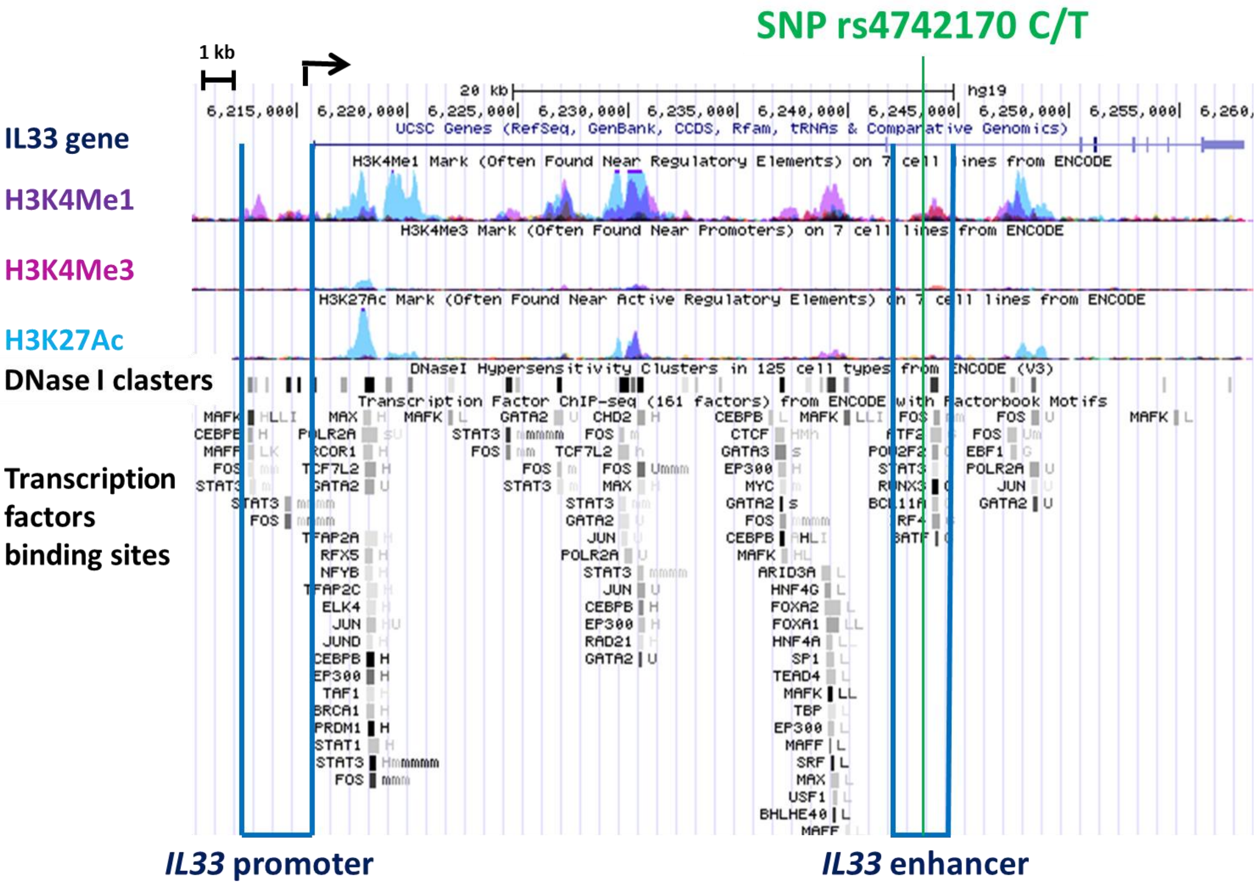

Supplementary Figure S1. A scheme of *IL33* gene (as displayed in UCSC Genome Browser) with maps of histone modifications, DNase I hypersensitivity clusters and predicted transcription factors binding sites

Supplementary Table S1.

Primers for amplification of IL33 promoter, point mutagenesis in IL33 promoter	
Prom fw	GCGAAGCTTACCATTGAGTACAACCAGAA
Prom rev	AATCCATGGTATTCAGTCTTACCTTGTGA
SNP replace Promoter fw	TTTAATAGTTACGAGAGCAT
SNP replace Promoter rev	ATGCTCTCGTAACTATTAAA
CREB1 site mutation A fw	TTTAATAGCCCCAAGAGCAT
CREB1 site mutation A rev	ATGCTCTTGGGGCTATTAAA
CREB1 site mutation G fw	TTTAATAGCCCCGAGAGCAT
CREB1 site mutation G rev	ATGCTCTCGGGGCTATTAAA

Primers for amplification of fragments for pull-down assay	
Pull-down fw	GAAAGGCAGATCAGGAGAGA
Pull-down rev	TTCATTCCCACAACACCGAT
Pul-down control fw	TGATCTCGAACTCCTGACCT
Pull-down control rev	GTGCTGTGATTACAGGTGTG

Primers for real-time RT-PCR	
CREB1 fw	ACCACCGGTAACTAAATGACCA
CREB1 rev	CCCATTGGGCAGCTGTACT
β-actin fw	TGCGTGACATTAAGGAGAAG
β-actin rev	GTCAGGCAGCTCGTAGCTCT

siRNAs for CREB1 knockdown	
siRNA sense	GGAGGAGAGUCGCUACCUAtt
siRNA antisense	UAGGUAGCGACUCUCCUCCtg
Scrambled sense	GGUCGGCACACGUGAUGAAtt
Scrambled antisense	UUCAUCACGUGUGCCGACCtg
